# Elderly individuals living by themselves: knowledge and measures to prevent the novel coronavirus*

**DOI:** 10.1590/1518-8345.4675.3383

**Published:** 2020-11-06

**Authors:** Darlene Mara dos Santos Tavares, Nayara Gomes Nunes Oliveira, Gianna Fiori Marchiori, Mariana Silva Freitas Guimarães, Lenniara Pereira Mendes Santana

**Affiliations:** 1 Universidade Federal do Triângulo Mineiro, Departamento de Enfermagem em Educação e Saúde Comunitária, Uberaba, MG, Brazil.; 2 Scholarship holder at the Conselho Nacional de Desenvolvimento Científico e Tecnológico (CNPq), Brazil.; 3 Universidade Federal do Triângulo Mineiro, Uberaba, MG, Brazil.; 4 Scholarship holder at the Coordenação de Aperfeiçoamento de Pessoal de Nível Superior (CAPES), Brazil.

**Keywords:** Aged, Health of the Elderly, Aging, Severe Acute Respiratory Syndrome, Coronavirus Infections, Geriatric Nursing, Idoso, Saúde do Idoso, Envelhecimento, Síndrome Respiratória Aguda Grave, Infecções por Coronavírus, Enfermagem Geriátrica, Anciano, Salud del Anciano, Envejecimiento, Síndrome Respiratorio Agudo Grave, Infecciones por Coronavirus, Enfermería Geriátrica

## Abstract

**Objective:**

to describe the occurrence of COVID-19 and the health services used by elderly individuals living by themselves; identify the knowledge held by elderly individuals regarding the transmission, signs and symptoms of COVID-19, as well as factors associated with poor knowledge of preventive measures according to sociodemographic and clinical variables.

**Method:**

cross-sectional survey conducted by telephone or mobile with 123 elderly individuals living by themselves in the Health Macro-Region of *Triângulo Sul* in the state of Minas Gerais, Brazil. Descriptive analysis was performed along with bivariate and multiple linear regression (*p*<0.05).

**Results:**

most elderly individuals did not present COVID-19 signs and symptoms (97.5%), were aware of how it is transmitted (86.6%), and of its signs and symptoms (90.8%). The elderly individuals were familiar with four preventive measures on average. After social distancing began, 85.7% of them left home and implemented three preventive measures on average, the most frequent of which was the use of face masks (99.0%). Being a man (*p*=0.001), 80 years old or older (*p*=0.045), and having fewer years of schooling (*p*=0.010) were associated with having less knowledge regarding the COVID-19 preventive measures.

**Conclusion:**

the elderly individuals were knowledgeable on COVID-19, but did not implement all the preventive measures. Male elderly individuals living by themselves with a low educational level are more vulnerable to COVID-19.

## Abstract (pt)

**Objetivo:**

descrever a ocorrência da COVID-19 e o local de atendimento entre idosos que moram sozinhos; identificar o conhecimento dos idosos sobre a transmissão, sinais e sintomas e medidas preventivas da COVID-19 e verificar os fatores associados ao menor conhecimento das medidas preventivas segundo variáveis sociodemográficas e clínicas.

**Método:**

inquérito telefônico e transversal desenvolvido com 123 idosos que moram sozinhos na Macrorregião de Saúde do Triângulo Sul no Estado de Minas Gerais. Realizaram-se análises: descritiva, bivariada e regressão linear múltipla (*p*<0,05).

**Resultados:**

a maioria dos idosos não apresentou sinais e sintomas da COVID-19 (97,5%), conhecia sua forma de transmissão (86,6%) e os sinais e sintomas (90,8%). Os idosos conheciam, em média, quatro medidas preventivas da COVID-19. Após o início do distanciamento social, 85,7% saíram do domicílio e realizaram, em média, três medidas preventivas, sendo mais frequente o uso de máscaras (99,0%). O menor conhecimento sobre as medidas preventivas da COVID-19 associou-se ao sexo masculino (*p*=0,001), faixa etária de 80 anos ou mais (*p*=0,045) e menor escolaridade (*p*=0,010).

**Conclusão:**

os idosos possuíam conhecimento sobre a COVID-19, porém, não realizavam todas as medidas preventivas. Além disso, idosos que moram sozinhos do sexo masculino, longevos e com baixa escolaridade estão mais vulneráveis à COVID-19.

## Abstract (es)

**Objetivo:**

describir la incidencia de COVID-19 y el local de atención entre los adultos mayores que viven solos; identificar el conocimiento de los adultos mayores sobre la trasmisión, señales y síntomas y medidas preventivas de COVID-19 y comprobar los factores asociados al menor conocimiento de las medidas preventivas según variables sociodemográficas y clínicas.

**Método:**

encuesta telefónica y trasversal, desarrollada con 123 adultos mayores que viven solos en la Macroregión Sanitaria del Triângulo Sul en el estado de Minas Gerais. Fueron aplicados análisis: descriptivo, bivariado y regresión lineal múltiple (*p*<0,05).

**Resultados:**

la mayoría de los adultos mayores no presentaron señales y síntomas de COVID-19 (97,5%), conocía su forma de trasmisión (86,6%) y los señales y síntomas (90,8%). Los adultos mayores conocían en promedio cuatro medidas preventivas de COVID-19. Tras el inicio del distanciamiento social, el 85.7% salió del hogar y practicó en promedio tres medidas de prevención, siendo más frecuente el uso de mascarillas (99,0%). El menor conocimiento sobre las medidas preventivas de COVID-19 se asoció con el sexo masculino (*p*=0,001), el rango de edad de 80 años o más (*p*=0,045) y menor escolaridad (*p*=0,010).

**Conclusión:**

los adultos mayores tenían conocimiento sobre la COVID-19, pero no aplicaban todas las medidas preventivas. Además, los adultos mayores que viven solos, mayores y con baja escolaridad son más vulnerables a la COVID-19.

## Introduction

COVID-19, from the term “*coronavirus disease* 2019”, is an acute respiratory disease, caused by the novel coronavirus (SARS-CoV-2)^(^ 1 ^)^, which was declared Public Health Emergency of International Concern and characterized as a pandemic on March 11^th^, 2020^(^ 2 ^)^.

Its clinical management and appropriate treatment have not yet been fully recognized^(^ 1 ^)^. It is known, however, that the virus is highly transmissible and mainly spreads from person to person^(^ 3 ^)^. Transmission occurs through respiratory droplets released through the nose or mouth when an infected person coughs or sneezes^(^ 1 ^)^. The presence of the virus on objects or surfaces is another form of contagion; hence, people are at risk of becoming infected if they touch their eyes, nose or mouth after touching contaminated objectives or surfaces^(^ 1 ^)^.

The clinical condition of COVID-19 in most cases is similar to other respiratory infections: fever (≥37.8ºC), running nose, dry cough, and fatigue^(^ 2 ^)^. Approximately 5% of the infected individuals develop the most severe form of the disease, with symptoms such as dyspnea and/or pulmonary bleeding, severe lymphopenia, and renal failure^(^ 4 ^)^.

Given the seriousness of the world situation and the need to decrease the spread of COVID-19 and flatten its epidemiological curve, preventive measures were adopted such as hand sanitation, respiratory etiquette, cleaning and disinfection of surfaces and objects, and social distancing^(^ 2 ^)^. Delay in adopting these measures resulted in an epidemiological curve with high mortality rates in Brazil, Italy, Spain, and the United States of America, even surpassing China^(^ 2 ^)^.

Due to the physiological changes inherent to the human aging process, which compromise the immunological system, and the greater number of complications accruing from chronic diseases at old age, the elderly are more susceptible to the more severe manifestations of COVID-19 and, consequently, death^(^ 5 ^-^ 6 ^)^. Additionally, sociodemographic and economic characteristics, such as advanced age; low educational and income levels; and single-person households, favor the greater vulnerability of elderly individuals to COVID-19. These factors may interfere in the access to information and, consequently, knowledge and implementation of measures to prevent infection by the novel coronavirus^(^ 7 ^-^ 10 ^)^.

Coupled with these, there has been an increase in the number of elderly individuals living by themselves^(^ 11 ^)^. In Brazil, 15.7% of individuals aged 60 years old or older live alone^(^ 12 ^)^; with the South (15.9%) and Southeast (15.7%) presenting the highest percentages^(^ 11 ^)^. Single-person households may be seen as an accomplishment considering that older people can enjoy greater privacy and independence as they age^(^ 11 ^)^, however, elderly individuals living by themselves may become more vulnerable and deprived of social support when facing health problems^(^ 11 ^,^ 13 ^)^. Additionally, access to information depends on the context in which individuals live^(^ 7 ^-^ 8 ^)^ and knowledge regarding preventive measures is one of the crucial factors to prevent the contagion and spread of the novel coronavirus^(^ 1 ^)^.

In this context, the identification of factors related to poor knowledge regarding the COVID-19 preventive measures among the elderly can support the practice of health workers, especially nurses, to combat the novel coronavirus. Nonetheless, even though this is a relevant topic, few scientific studies are addressing the knowledge about measures to prevent COVID-19 and their implementation among elderly individuals living by themselves. Studies addressing the elderly population has investigated the impact of the COVID-19 outbreak in long-term residential facilities for the elderly^(^ 14 ^-^ 15 ^)^; the knowledge held by adult and elderly individuals living in the community and their behavior in the face of COVID-19^(^ 7 ^-^ 8 ^)^; the beliefs regarding the COVID-19 pandemic held by the population in a Brazilian state^(^ 16 ^)^; the consequences of social isolation in the lives of elderly individuals^(^ 13 ^,^ 17 ^)^; and the clinical characteristics and prognosis factors among COVID-19 patients aged 60 years old or older^(^ 5 ^-^ 6 ^)^.

Therefore, the objective was to describe the occurrence of COVID-19 and the health services sought by elderly individuals living alone; identify the knowledge these individuals have concerning the COVID-19 transmission, signs and symptoms and preventive measures, and verify factors associated with poor knowledge of preventive measures according to sociodemographic and clinical variables.

## Method

A cross-sectional and analytical study with a quantitative approach using a telephone survey was conducted in May 2020 in the Health Macro-Region of *Triângulo Sul* in the state of Minas Gerais, which is subdivided into three micro-regions: Araxá and Uberaba, each with eight cities, and Frutal/Iturama with 11 cities (Table 1).

**Table 1:** Number of elderly individuals living by themselves with a landline telephone or a mobile, according to health micro-regions in the *Triângulo Sul*, MG, Brazil, 2020

Micro-regions in the Southern Triangle of Minas Gerais	Elderly individuals w/ telephone/mobile	Elderly individuals interviewed
Araxá	58	33
Frutal/Iturama	55	29
Uberaba	166	61
Total	279	123

The study population was composed of individuals living by themselves who had been interviewed in the study *“*Dependence to perform activities of daily living, frailty, and the use of health services among elderly individuals from the *Triângulo Sul* in the State of Minas Gerais”, which used multistage cluster sampling and interviewed 1,635 elderly individuals living in the community in the Health Macro-Region of the *Triângulo Sul* in the state of Minas Gerais, Brazil.

Individuals aged 60 years old or older; residing in the urban area of the Health Macro-Region of the *Triângulo Sul* in the state of Minas Gerais; living by themselves; and having a landline telephone or a mobile phone, were included in the study. Those who no longer lived alone at the time of the interview (n=12) or presented impaired hearing (n=2) were excluded. A total of 279 individuals in the database met the criteria and composed the initial sample. Those who were not found after three attempts (n=134) or refused to participate (n=8) were considered losses. Hence, 123 elderly individuals composed this study’s final sample (Table 1).

Sociodemographic data and morbidities were obtained by applying a structured questionnaire developed by the Collective Health Research Group.

The instrument used to collect data concerning knowledge of the COVID-19 was developed by the authors based on information and recommendations of the Ministry of Health^(^ 1 ^)^. It contained questions addressing the occurrence of COVID-19, health services used, forms of transmission, signs and symptoms, and preventive measures. A pilot study was conducted by telephone with ten elderly individuals who were the researchers’ acquaintances and lived by themselves, to test, assess, revise, and refine the instrument.

The main COVID-19 preventive measures considered were: using face masks; handwashing with water and soap and/or sanitizing with alcohol gel; protecting the nose and mouth with a tissue or arm when coughing or sneezing; not touching the eyes, mouth or nose; leaving home only when necessary; keeping a minimum distance of 2 meters from other people; not kissing or handshaking; not receiving visits; and removing the shoes whenever returning home and sanitizing them, if possible; removing clothes and putting them in a plastic bag or laundering them; leaving purse, wallet, and keys in a box at the home entrance; keeping the home clean and ventilated; cleaning objects with water and soap or 70% alcohol; washing foods and products when getting back from the grocery store; getting enough sleep and eating healthy; and not sharing personal utensils such as cutlery, towels, plates or glasses^(^ 1 ^)^.

The variables included: sex (female or male); age range in complete years (60├70; 70├80; 80 or more); marital status (single, widowed, separated, or divorced); schooling years (none; 1├5; 5 or more); individual monthly income in terms of times of minimum wage (no income; ≤1; >1); number of morbidities (0; 1├5; 5 or more); presence of signs and symptoms of COVID-19 (yes; no); health service (primary health care unit; emergency service; private medical office; referral hospital; others); knowledge about COVID-19 (yes; no), forms of transmission (yes; no) and signs and symptoms (yes; no); source of information (TV; radio; family members; friends and internet); whether has left home during the quarantine (yes; no); whether implemented preventive measures when leaving home (yes; no); and average number of preventive measures known and implemented.

Data were collected among elderly individuals living by themselves in May 2020 by telephone. Experienced interviewers were previously trained on ethical issues and on how to conduct the interviews.

The interviews were recorded in an electronic database in Excel^®^ and then revised by the supervisors. Whenever necessary, the files were sent back to the interviewers to complement the information. After this stage, all interviewers’ databases were consolidated into a single one and transferred to Statistical Package for Social Sciences version 22.0 for analysis.

The absolute and relative frequencies of categorical variables were verified and the means and standard deviations were found for the quantitative variables. A preliminary bivariate analysis was performed using Student’s t-test to verify the factors associated with less knowledge concerning COVID-19 preventive measures. The variables of interest that met the criterion (*p*≤0.10) were included in the multiple linear regression model (*p*<0.05). The outcome was the average number of COVID-19 preventive measures the elderly participants were familiar with and the predictive variables were: sex, age range, monthly individual income; schooling; and number of morbidities. The following variables were dichotomized to perform the bivariate and multiple linear regression analyses: age range (60├80 years old; 80 years old or older); monthly individual income, times of minimum wage (>1; ≤1); and number of morbidities (0├5; 5 or more). The schooling variable was used in its quantitative form (complete years of schooling).

The Institutional Review Board approved the study project on May 13^th^, 2020 under protocol No. 4.026.689. The interviews were conducted after the participants’ consent according to the guidelines established by Resolution 510/16, Ministry of Health^(^ 18 ^)^. After the interviews, the participants received instructions regarding preventive measures against COVID-19.

## Results

Most elderly individuals were women (69.9%), aged 70├80 years old (45.5%), widowed (67.5%), had 1├5 years of schooling (54.5%), a monthly individual income >1 times the minimum wage (56.3%); and 1├5 morbidities (69.9%).

A larger portion of the participants did not present signs and symptoms of COVID-19 (97.5%) and those who did (n=2) reported dry cough; running nose and fatigue (50.0%); sought a private health service (50.0%), and tested negative for COVID-19 (50.0%).

A total of 96.3% of the elderly individuals were familiar with COVID-19 and their most frequent source of information was TV (96.6%); followed by radio (28.6%); family members (25.2%); friends (15.1%); and Internet (10.9%).

Most individuals were aware of how COVID-19 (86.6%) is transmitted and also about its signs and symptoms (90.8%). The form of transmission most frequently reported was handshaking (79.6%), followed by sneeze (72.8%), contaminated objects and surfaces (70.9%), and saliva droplets (33.0%). Regarding signs and symptoms, the most frequently reported were fever (73.1%); difficulty to breath (59.2%); sore throat, body ache and headache (52.8%); dry cough (26.8%); fatigue (13.9%); running nose (12.0%); loss of taste and smell (10.2%), and diarrhea (4.6%).

A total of 98.3% of the elderly participants were aware of the COVID-19 preventive measures, most frequently the use of face masks and hand sanitizing with soap and water or alcohol gel (Table 2). Among the 17 preventive measures assessed, the participants reported 3.98 (±1.76) on average.

**Table 2:** Frequency distribution of the COVID-19 preventive measures reported by the elderly individuals living by themselves in the Health Macro-Region of the *Triângulo Sul*, MG, Brazil, 2020

COVID-19* preventive measures	n=117	%
Wearing face masks	102	87.2
Handwashing with water and soap	100	85.5
Hand sanitizing with alcohol gel	84	71.8
Staying home	81	69.2
Keeping a minimum distance of 2 meters between people	37	31.6
Not hugging, kissing, or handshaking	19	16.2
Not receiving visits	19	16.2
Taking the shoes off when returning home and sanitizing them, if possible	8	6.83
Not touching eyes, nose or mouth	8	6.83
Taking the clothes off when returning home and putting them in a plastic bag or washing them	7	6.0
Keeping the home clean and ventilated	6	5.1
Cleaning objects with water and soap or sanitizing them with alcohol at 70%	5	4.3
Protecting the nose and mouth with a tissue or arm when coughing or sneezing	4	3.4
Washing foods and cleaning products when returning from the grocery store	4	3.4
Sleeping well and eating healthy	2	1.7
Not sharing personal objects such as cutlery, towels, plates, or glasses.	1	0.8
Whenever returning home, leaving the purse, wallet, and keys in a box at the home entrance	0	0.0

* Coronavirus Disease 2019

After social distancing began, 85.7% of the elderly individuals left home and followed 2.58 (±1.91) preventive measures on average. Of these, 98.0% implemented preventive measures, mainly the use of face masks and handwashing with soap and water (Table 3).

**Table 3:** Frequency distribution of COVID-19 preventive measures implemented when elderly individuals living by themselves at home, Health Macro-Region of *Triângulo Sul*, MG, Brazil, 2020

COVID-19* preventive measures	n=100	%
Wearing face masks	99	99.0
Handwashing with water and soap	72	72.0
Avoiding crowded places	40	40.0
Keeping a minimum distance of 2 meters between people	37	37.0
Taking the shoes off when returning home and sanitizing them, if possible	18	18.0
Taking the clothes off when returning home and putting them in a plastic bag or washing them	19	19.0
Avoiding crowded transportation	15	15.0
Not hugging, kissing or handshaking	10	10.0
Cleaning objects with water and soap or sanitizing them with alcohol at 70%	3	3.0
Whenever returning home, leaving the purse, wallet, and keys in a box at the home entrance	0	0.0

* Coronavirus Disease 2019

The following variables met the criterion (*p*≤0.10) in the bivariate analysis and were included in the multiple linear regression final model: sex, age range, schooling, monthly individual income, and number of morbidities (Table 4).

**Table 4:** Bivariate analysis concerning knowledge of COVID-19 preventive measures according to sociodemographic variables and number of morbidities among elderly living by themselves in the Health Macro-Region of the *Triângulo Sul*, MG, Brazil, 2020

Variables	Mean	Standard deviation	p*
**Sex**			
Male	3.24	1.49	0.001
Female	4.29	1.78	
**Age range**			
80 years old or older	3.31	1.71	0.004
60├80 years old	4.29	1.71	
**Monthly individual income**			
≤ 1	3.74	1.50	0.087
>1	4.26	1.90	
**Morbidities**			
0├5	3.62	1.70	0.089
5 or more	4.12	1.78	

* *p*≤0.10

Being a man, 80 years old or older, and having a lower level of education were associated with poor knowledge of COVID-19 preventive measures (Table 5).

**Table 5:** Final multiple linear regression model of knowledge concerning COVID-19 preventive measures among elderly individuals living by themselves in the Health Macro-Region of the *Triângulo Sul*, MG, Brazil, 2020

Variables	β	p*
**Sex**		
Female	1	
Male	0.283	**0.001**
**Age range**		
60├80 years old	1	
80 years old or older	0.206	**0.045**
**Monthly individual income**		
> 1	1	
≤ 1	0.105	0.231
**Schooling (complete years)**	0.224	**0.010**
**Morbidities**		
5 or more	1	
0├5	0.100	0.242

* *p*<0.05; 1- reference category

## Discussion

The higher percentage of women found in this study is in line with the reports of Brazilian studies^(^ 11 ^)^. One study conducted in a city in Minas Gerais verified that most elderly individuals in the group who lived by themselves were women^(^ 19 ^)^. The predominance of women among elderly individuals may be explained by the higher life expectancy among women in comparison to men, which is currently 80.25 years old in Brazil^(^ 20 ^)^.

The larger percentage of widows may be related to the predominance of women and their life expectancy, though women are also more likely to live by themselves than remarrying or being the head of the family^(^ 21 ^)^.

Regarding age, similar results were found in a Brazilian study, in which a larger number of individuals aged 75 years old or older lived alone compared to younger individuals (*p*<0.001)^(^ 11 ^)^. A Brazilian regional study, however, verified that most elderly individuals living by themselves were 60 to 69 years old^(^ 19 ^)^. Individuals in advanced age living by themselves may face problems to use health services and perform daily tasks, which may become more difficult in the absence of family members^(^ 19 ^)^.

Similar to these findings, a study verified that most elderly individuals living by themselves had from one to four years of schooling^(^ 19 ^)^. Divergent data were found in a Brazilian survey in which the percentage of elderly individuals in single-person households (16.9%) had from eight to ten years of schooling^(^ 11 ^)^. A low educational level negatively affects self-care behavior^(^ 9 ^)^ due to difficulties to access and assimilate information^(^ 10 ^)^.

Concerning monthly individual income, most of the participants living by themselves in a city in the interior of Minas Gerais received from one to three times the minimum wage^(^ 19 ^)^, a piece of information that is in line with this study’s findings.

In times of COVID-19 pandemic, nurses should pay attention to the sociodemographic characteristics of elderly individuals living by themselves, considering that advanced age and low levels of education and income may hinder the acquisition of knowledge and adherence to preventive measures, as well as limit access to health services and decrease adherence to social isolation.

A similar result was found in a Brazilian study, which reports that 52.4% of the elderly individuals living by themselves presented two or more morbidities^(^ 11 ^)^. This is a relevant aspect when assessing elderly individuals as the scientific literature describes that people with chronic diseases are more susceptible to the more severe manifestations of the COVID-19^(^ 5 ^)^. The reason is that immunological functions may decrease with age and in the face of comorbidities, favoring the more severe forms of the COVID-19^(^ 5 ^-^ 6 ^)^.

International studies report that the main signs and symptoms presented by individuals diagnosed with COVID-19 include fever, cough, fatigue, and body ache^(^ 5 ^,^ 22 ^-^ 23 ^)^, the same symptoms presented by this study’s participants. Note that a few participants presented signs and symptoms, which makes sense considering the current COVID-19 panorama in the state of Minas Gerais, with smaller numbers of confirmed and suspected cases and deaths than in the remaining Brazilian states^(^ 24 ^)^.

Despite the number of elderly individuals presenting signs and symptoms of COVID-19 in this study, this age group presents a less coordinated, less efficient and slower immune response, making them more susceptible to emerging infections such as the new coronavirus^(^ 25 ^)^. Additionally, some elderly individuals may feel intimidated or discouraged to report symptoms of this disease because society addresses aspects of aging negatively, mainly emphasizing its limitations^(^ 26 ^)^; even considering the provision of priority health care to elderly individuals in comparison to younger individuals unnecessary, which may negatively impact their perception.

In this context, nurses should screen elderly individuals with signs and symptoms of the COVID-19 and verify the need to perform a physical assessment in person and monitor these individuals’ conditions, instructing about the need to comply with the quarantine period and having their condition reassessed until the symptoms disappear completely.

Most of the individuals addressed in this study reported they acquired knowledge about COVID-19 by watching TV. This finding is in line with a Brazilian study addressing the behavior of the elderly, in which 31.2% of those living by themselves watched TV for five or more hours a day. The prevalence of this activity was 40.0% greater among those living by themselves than among elderly individuals who lived with other people^(^ 11 ^)^. Even though watching TV for long periods is sedentary behavior, in this pandemic, it was a considerable source of information among those living by themselves. Note that this behavior may facilitate the acquisition of information concerning COVID-19, and objectively and reliable information can improve the perceived effectiveness of COVID-19-oriented behaviors, positively affecting the adoption of measures prescribed to fight the pandemic^(^ 9 ^)^.

The knowledge of the participants regarding how COVID-19 is transmitted is consonant with studies reporting the permanence of the virus on surfaces for hours to days^(^ 27 ^)^ when expelled through cough or sneeze^(^ 27 ^-^ 28 ^)^, and the need to avoid handshaking and touching contaminated surfaces^(^ 27 ^)^, considering that a single droplet may contain an infectious dose^(^ 27 ^)^.

An international study reports that 71.7% of elderly individuals had knowledge of three symptoms of COVID-19^(^ 7 ^)^, which is in line with the mean identified in this study. The infectious condition with the onset of fever is the clinical symptoms more commonly identified in COVID-19^(^ 29 ^-^ 30 ^)^, considered essential to screening the disease^(^ 25 ^)^, which is corroborated by the elderly individuals’ reports. The period in which fever manifests, however, is unknown^(^ 30 ^)^. Elderly individuals may present temperatures below that presented by other population groups^(^ 25 ^)^, which shows the need for monitoring and frequently assessing individuals’ thermal curve and associate it with other complaints of an infectious nature that may be related to COVID-19, such as respiratory symptoms and body ache.

Because it is considered an acute respiratory syndrome, symptoms such as difficulty breathing are commonly identified in the scientific literature^(^ 25 ^)^, which is similar to what the individuals addressed in this study recognized in themselves, that is, dyspnea may be confounded with some other comorbidity such as heart failure, rather than recognized as a new complaint or suspicion of COVID-19^(^ 25 ^)^. Additionally, pulmonary changes such as pneumonia accruing from severe infection may exacerbate respiratory symptoms and require hospitalization^(^ 25 ^)^, leading to greater vulnerability and decreasing the likelihood of survival, especially among the elderly.

Additionally, people may complain of body ache, fatigue, and malaise^(^ 5 ^,^ 29 ^)^. One scoping review and meta-analysis identified that 36% of the patients reported body ache, 12% reported headache, and 10% sore throat^(^ 30 ^)^. When the percentages are totaled, the results are similar to those found in this study, in which 52.8% of the elderly participants living by themselves reported being aware of these COVID-19 symptoms.

Nurses, together with the multiprofessional team, should encourage elderly individuals to perform self-care and be attentive to the emergency of signs and symptoms related to COVID-19, emphasizing the importance of reporting signs and symptoms to the primary health care team to assess the situation and follow-up of government protocols. The infection by the novel coronavirus among elderly individuals usually presents atypical manifestations, which hinder its early identification and control, so that preventive measures are key to manage and control COVID-19^(^ 25 ^)^.

One international study verified that 69.8% of the elderly individuals were aware of three preventive measures to combat COVID-19^(^ 7 ^)^, which is below the number found in this study. Nonetheless, even though most of the elderly participants were familiar with the preventive measures, they reported four measures on average, which is a low number if we consider the 17 measures recommended by the Ministry of Health^(^ 1 ^)^.

The use of face masks is a measure adopted worldwide to prevent and control COVID-19, which has been widely reported by scientific studies^(^ 27 ^,^ 31 ^-^ 32 ^)^ and is in line with this study’s findings. The purpose of wearing masks is to protect all individuals, both healthy individuals and those diagnosed with the disease, to control the transmission and avoid new contaminations^(^ 33 ^)^. The implementation of this practice alone is not sufficient to protect and control the disease, that is, it must be associated with other measures, such as hand washing and the use of alcohol gel^(^ 33 ^)^.

Handwashing, one of the COVID-19 preventive measures, reflects the knowledge reported by elderly individuals living by themselves. The main form the virus is spread is from person to person through droplets, followed by aerosols^(^ 25 ^)^, and hands are the main route of cross-contamination^(^ 27 ^)^. The virus may remain on surfaces and in the environment for different periods, ranging from hours to days^(^ 34 ^)^. For this reason, besides being a low-cost practice, hand sanitation is highly effective and is considered one of the most relevant measures to prevent COVID-19^(^ 27 ^)^.

The elderly individuals living by themselves also reported the use of gel alcohol as a preventive measure, emphasized by health and government organizations since the beginning of the COVID-19 pandemic^(^ 33 ^,^ 35 ^)^. Nonetheless, for microorganisms to be inactivated and levels of infection and transmission to decrease, the alcohol needs to present a concentration between 62% and 71%^(^ 36 ^)^. Its use is necessary in environments outside the home where water and soap are not readily available, whenever one has physical or close contact with people, objects, or surfaces that may be contaminated.

Note that social distancing was not a preventive measure most frequently reported by the elderly individuals, which is opposed to the expressive dissemination in the media and scientific studies of its needs and positive effects in the control of the transmission of COVID-19^(^ 32 ^,^ 37 ^)^.

One study conducted in the state of Ceará, Brazil reports that elderly people partially adhered to social distancing and received visits (62.5%)^(^ 16 ^)^. The same was found in this study, in which most elderly individuals left their homes during the time social distancing was recommended. Even though the Brazilian authorities emphasize social distancing, specifically for the groups most susceptible to develop the severe form of the disease^(^ 1 ^-^ 2 ^,^ 26 ^,^ 32 ^,^ 37 ^)^, one needs to consider and understand the reasons that lead these individuals to leave their homes.

Individuals living by themselves and without social support need to run errands to meet their needs, such as: go to the grocery store and pay bills, among others, while the information they received may not have been sufficient to convince them of the need to adhere to social distancing. Emotional aspects such as loneliness also need to be considered in addition to beliefs and values. One Brazilian study reports that elderly individuals believed that the pandemic would be less severe in Brazil than in other countries^(^ 16 ^)^. Note that the way individuals respond to situations, such as a pandemic, vary according to their engagement in preventive care, and how they address problems and search for solutions^(^ 9 ^)^. Hence, in this context in which multiple factors may interfere in the low adherence of elderly individuals to social distancing, nurses need to identify the motivations of these individuals and establish joint actions to decrease the likelihood of them being contaminated by the COVID-19, such as: having instrumental and emotional support provided by family members and friends and identifying activities to spend time at home, among others.

The average number of preventive measures adopted by the individuals addressed in this study when leaving home was below the number of measures they reported to be familiar with. The most widely known measures, the use of face masks and handwashing with soap and water, were the ones most frequently adopted, followed by avoiding crowded places.

The use of masks was also verified in a systematic review with meta-analysis, which reports an association between its use and the protection of healthy individuals at home and whenever social interaction is necessary^(^ 38 ^)^. Its use involves a series of measures though, necessary to keep it viable, without becoming an object of new contaminations. For this reason, for a mask to function as a mechanical barrier against the novel coronavirus^(^ 35 ^)^, one should follow recommendations on how to put and remove it, how to sanitize it, and regarding the period it remains viable, up to its discard^(^ 35 ^)^.

The use of masks is coupled with hand sanitation, the second preventive measure the elderly individuals reported, which is consistent with the literature^(^ 27 ^,^ 33 ^,^ 35 ^)^. Nonetheless, even when implementing the aforementioned measures, agglomerations should be avoided^(^ 33 ^-^ 35 ^)^.

Even though these measures are recommended^(^ 33 ^-^ 35 ^)^ to ensure greater protection against transmission, one has to implement various measures together^(^ 27 ^,^ 31 ^-^ 32 ^)^, such as those necessary to implement when returning home, which few participants reported.

Considering that behavioral change depends on the context one is inserted in, and is characterized by the unpredictability of different sociodemographic and economic characteristics^(^ 39 ^)^, these findings reinforce the need for nurses to implement educational actions together with elderly individuals by using effective communication.

The association identified in this study between being a man and having poor knowledge of measures to prevent COVID-19 is in line with the reports of studies conducted in the United States with adults and elderly individuals living in the community in which elderly men presented less knowledge of preventive measures than women and adult individuals (*p*<0.001)^(^ 7 ^-^ 8 ^)^.

The little knowledge of COVID-19 preventive measures among the elderly men may be related to the fact that they do not value self-care; are little concerned with their health, and have difficulties sharing their feelings and verbalizing their needs^(^ 40 ^)^. This context may also affect the mortality rates caused by COVID-19, which are higher among male elderly individuals^(^ 41 ^-^ 43 ^)^. In Italy, 83.0% of the deaths occurred among 60-year-old or older individuals; 80% of whom were men^(^ 42 ^)^. The last Epidemiological Bulletin published on May 23^rd^, 2020 reports that 69.4% of the deaths caused by COVID-19 in Brazil were among 60-year-old or older individuals, while 60.2% of them were men^(^ 43 ^)^. According to the Epidemiological Report of the COVID-19 pandemic, published on June 6^th^, 2020, 72.7% of the deaths in Minas Gerais were among the elderly, 53.0% of whom were men^(^ 24 ^)^.

In this context, educational actions in the nursing field should be planned to reach elderly men living by themselves. In addition to educational practices within the healthcare network^(^ 44 ^)^, nurses’ competencies such as preventive actions and the early detection of infections by the novel coronavirus should be implemented during gerontological nursing consultations.

In line with this study, one international study addressing adult and elderly individuals in the community reports that individuals of advanced age and those living by themselves presented a lower level of knowledge of COVID-19 (*p*<0.001)^(^ 7 ^)^, showing that older individuals are more vulnerable to the infection caused by the novel coronavirus^(^ 6 ^)^. These circumstances reinforce the need for actions and specific interventions for 80 years-old or older individuals living by themselves, as they may need more complex care with greater investment in health, like in the case of the infection caused by the novel coronavirus^(^ 7 ^)^.

The bond established between long-lived individuals and health workers was associated with preventive measures to combat COVID-19 (*p*=0.008) in a study conducted in the state of Ceará, Brazil^(^ 16 ^)^. This bond contributes to elderly individuals being more pro-active in the health-disease continuum and also promote the delivery of comprehensive health care^(^ 45 ^)^. The primary health care policy provides for the establishment of bonding and co-accountability between health teams and the population, ensuring the continuity of actions as well as establishing the service as a reference for these individuals^(^ 46 ^)^. From this perspective, bonding emerges as a fundamental element to favor the adherence of elderly individuals who live by themselves to the COVID-19 preventive measures.

One study conducted in the United States also found that elderly individuals (*p*<0.001), as well as those living alone (*p*<0.001), and with a low educational level (*p*<0.001), presented the worst level of knowledge concerning COVID-19^(^ 7 ^)^. The low educational level of the elderly individuals may be considered a risk factor for the dissemination of viral infections and death^(^ 7 ^,^ 16 ^)^, considering that it may negatively influence the way elderly individuals understand and perform self-care^(^ 47 ^)^. Additionally, a low educational level may be associated with an individual’s social status, suggesting that lifestyle, living conditions, and knowledge concerning COVID-19 influence its prognosis^(^ 7 ^,^ 16 ^)^. Therefore, less educated elderly individuals would be more prone to the infection caused by the novel coronavirus because they use crowded public transportation and have poor access to medical resources^(^ 16 ^)^.

This context represents a challenge for health workers, especially nurses providing care to elderly individuals living by themselves with a low educational level. For this reason, strategies that favor the knowledge and adherence to measures that prevent the spread and transmission of COVID-19 are needed^(^ 16 ^)^. Nurses should attempt to use efficient communication with these individuals, using clear and objective language when addressing the care necessary to prevent this disease. Communication in the nursing scope is an essential strategy in the care provided to elderly individuals because it encourages individuals to trust health workers and expose their needs, promoting positive interaction between care recipients and nurses^(^ 48 ^)^.

One of this study’s limitations refers to its cross-sectional design, which does not allow for the establishment of causal effects, and its sample, which was restricted to individuals who had a landline phone or a mobile. The results, however, contribute to the advancement of scientific knowledge, supporting the practice of nurses providing care in times of pandemic to elderly individuals living by themselves. This study’s findings reinforce the need for health actions promoting measures to prevent the COVID-19 infection among the elderly, paying attention and implementing strategies that are specific to elderly men, aged 80 years old or older, with a low educational level. Additionally, the results can also direct the practice of nurses working in the primary health care network and having greater contact with these individuals, to perform nursing actions intended to prevent the infection by the novel coronavirus.

## Conclusion

A higher percentage of elderly individuals living by themselves did not present any signs or symptoms of COVID-19 and were aware of its forms of transmission. The form of transmission most frequently reported was shaking hands and the most frequently reported symptom was fever.

The elderly participants reported knowledge of four preventive measures on average; while the use of masks prevailed. The implementation of three measures on average was reported by the elderly individuals when they left their homes though, the most frequent of which was the use of face masks.

Being an 80-year-old or older man and having a low educational level were associated with having less knowledge regarding the COVID-19 preventive measures.

These results can support the planning of social and health actions directed to the elderly population living alone in the face of the COVID-19 pandemic.
